# AR imposes different effects on *ZFHX3* transcription depending on androgen status in prostate cancer cells

**DOI:** 10.1111/jcmm.17125

**Published:** 2021-12-24

**Authors:** Xing Fu, Zhiqian Zhang, Mingcheng Liu, Juan Li, Jun A, Liya Fu, Chenyang Huang, Jin‐Tang Dong

**Affiliations:** ^1^ Department of Genetics and Cell Biology College of Life Sciences Nankai University Tianjin China; ^2^ Department of Human Cell Biology and Genetics School of Medicine Southern University of Science and Technology Shenzhen China

**Keywords:** androgen, AR, prostate cancer, protein degradation, transcription, ZFHX3

## Abstract

Both androgen receptor (AR) and the ZFHX3 transcription factor modulate prostate development. While AR drives prostatic carcinogenesis, ZFHX3 is a tumour suppressor whose loss activates the PI3K/AKT signalling in advanced prostate cancer (PCa). However, it is unknown whether ZFHX3 and AR are functionally related in PCa cells and, if so, how. Here, we report that in AR‐positive LNCaP and C4‐2B PCa cells, androgen upregulates *ZFHX3* transcription via androgen‐induced AR binding to the androgen‐responsive elements (AREs) of the *ZFHX3* promoter. Androgen also upregulated *ZFHX3* transcription in vivo, as castration dramatically reduced *Zfhx3* mRNA and protein levels in mouse prostates, and *ZFHX3* mRNA levels correlated with AR activities in human PCa. Interestingly, the binding of AR to one ARE occurred in the absence of androgen, and the binding repressed *ZFHX3* transcription as this repressive binding was interrupted by androgen treatment. The enzalutamide antiandrogen prevented androgen from inducing *ZFHX3* transcription and caused excess ZFHX3 protein degradation. In human PCa, *ZFHX3* was downregulated and the downregulation correlated with worse patient survival. These findings establish a regulatory relationship between AR and ZFHX3, suggest a role of ZFHX3 in AR function and implicate ZFHX3 loss in the antiandrogen therapies of PCa.

## 1 INTRODUCTION

Prostate cancer (PCa) is one of the most prevalent cancers among men.^1^, ^2^ The androgen/androgen receptor (AR) signalling is a driving force in the development and maintenance of normal prostates and prostatic carcinogenesis.^3^ Accordingly, androgen deprivation therapy (ADT) via surgical or chemical castration is an effective treatment for PCa.^4^, ^5^, ^6^ Unfortunately, most PCa patients undergoing ADT will eventually develop resistance to ADT, and their tumours progress to castration‐resistant prostate cancer (CRPC), which often becomes lethal. In CRPC, AR signalling is commonly reactivated via different mechanisms, including genetic alterations in the *AR* gene and unknown mechanisms.^7^, ^8^, ^9^ It is crucial to uncover additional molecular mechanisms for the reactivation of AR signalling in CRPC.

Zinc finger homeobox 3 (ZFHX3), also known as AT‐motif binding factor 1 (ATBF1), is a large transcription factor composed of 3703 amino acid residues containing four homeodomains, 23 zinc finger motifs and multiple other motifs.^10^ *ZFHX3* is frequently mutated in metastatic or high‐grade human PCa, and the mutations are mainly loss‐of‐function mutations.^11^, ^12^ Tissue‐specific deletion of *Zfhx3* induces mouse prostatic intraepithelial neoplasia (mPIN) and promotes prostatic tumorigenesis initiated by *Pten* deletion.^13^, ^14^ In cultured human prostate cancer cells, ZFHX3 is indispensable for oestrogen receptor beta (ERβ) to inhibit cell proliferation via MYC downregulation.^15^ These findings indicate that ZFHX3 plays a role in androgen‐driven prostatic development and that ZFHX3 is a tumour suppressor in PCa.

In PCa cells, activation of ERβ by diarylpropionitrile (DPN) upregulates ZFHX3, and ZFHX3 is crucial for ERβ to inhibit cell proliferation.^15^ In mammary epithelial cells, activation of ERα by oestrogen or PR by progesterone also upregulates ZFHX3 expression, and ZFHX3 is also involved in the functions of ER and PR.^16^, ^17^, ^18^ The upregulation is primarily at the transcriptional level. AR, ER and PR belong to the steroid receptor family and have structural similarities; AR and PR are even more similar than ER.^19^ In addition, androgens, oestrogens and progesterones share some synthetic routes.^20^ We, therefore, hypothesize that androgen/AR signalling also regulates ZFHX3 expression to regulate prostatic development and tumorigenesis.

This study examined the regulatory relationship between androgen/AR signalling and ZFHX3 in PCa cells. We demonstrated that the androgen/AR signalling upregulates the transcription of *ZFHX3* via the binding of AR to AREs of *ZFHX3* promoter in PCa cells, which appeared to occur in both mice and humans. Interestingly, binding to AR to another ARE of *ZFHX3* promoter occurred in the presence of androgen, and the binding repressed *ZFHX3* promoter activity. Furthermore, an inhibition of AR signalling by an AR antagonist prevented androgen‐induced *ZFHX3* transcription while causing excess degradation of ZFHX3 protein. These findings suggest that ZFHX3 is involved in AR function and implicate ZFHX3 loss in the treatment of PCa.

## 2 MATERIALS AND METHODS

### 2.1 Cell culture and RNA interference

Human PCa cell line LNCaP was purchased from ATCC, and C4‐2B was a gift from Dr. Leland Chung of Cedar Sinai Medical Center. Both cell lines were cultured in RPMI‐1640 medium supplemented with 10% foetal bovine serum (FBS; Gibco) in a humidified incubator (37°C and 5% CO_2_). For experiments involving the treatment of R1881 (Melonepharma, Dalian, China), cells were incubated in phenol red‐free medium containing 5% charcoal‐stripped FBS for 24 or 72 h, except the experiment involving a combined treatment of R1881 and enzalutamide (Beyotime), in which complete medium was used.

For gene silencing by RNA interference (RNAi), cells were transiently transfected with siRNAs using the Lipofectamine RNAiMAX reagent (Invitrogen). The siRNA sequence against human *AR* (siAR) was 5′‐CAAGGGAGGUUACACCAAA‐3′, validated in a previous study.^21^

### 2.2 Western blotting

Cultured cells were lysed in the cell lysis buffer for Western and IP from Beyotime. Cell lysates were subjected to 4% (for ZFHX3) or 10% (for all other proteins) sodium dodecyl sulphate‐polyacrylamide gel electrophoresis (SDS‐PAGE), and proteins were transferred to polyvinylidene fluoride (PVDF) membranes according to a standard protocol. The membranes were blocked with 5% nonfat milk for 30 min at room temperature and probed with primary antibodies overnight at 4°C. On the next day, the membranes were incubated with the secondary antibodies for 1 h at room temperature; and the WesternBright ECL HRP substrate (Advansta) was used with the Luminescent Image Analyzer (JUNYI, Beijing, China) to capture images.

Antibodies used in Western blotting included ZFHX3 (1:800, prepared in our previous study^22^), AR (1:2000, 5153s, Cell Signaling, Danvers, MA), Lamin B1 (1:1000, 13435s, Cell Signaling) and α‐tubulin (1:10000, T1699, Sigma, St Louis, MO).

### 2.3 RNA extraction and real‐time qPCR

Total RNA was extracted using the Eastep Super Total RNA Extraction Kit (Promega), and cDNA was synthesized with the Moloney murine leukaemia virus reverse transcriptase (Promega). Real‐time qPCR was performed with the 2x SYBR qPCR Mix (KT Life, Shenzhen, China) using the qTOWER3/G system (Analytik Jena).

Primer sequences were as follows: 5′‐GGTGGTCTCCTCTGACTTCAACA‐3′ (*GAPDH* forward), 5′‐GTTGCTGTGTAGCCAAATTCGTTGT‐3′ (*GAPDH* reverse), 5′‐TGTTCCAGATCGAGATGGGAAT‐3′ (*ZFHX3* forward), 5′‐CTTTCCCAGATCCTCTGAGGTTT‐3′ (*ZFHX3* reverse), 5′‐GTGTGTGGACCTCCATGTTATT‐3′ (*KLK3* forward), 5′‐TGCCCCATGACGTGATACCT‐3′ (*KLK3* reverse), 5′‐GATCTGGCACCACACCTTCT‐3′ (*Actb* forward), 5′‐GGGGTGTTGAAGGTCTCAAA‐3′ (*Actb* reverse), 5′‐AGAGCAAGAGGGCAGCGTCATC‐3′ (*Zfhx3* forward), 5′‐CGGTTCACGTCAGCGTTGCTATAC‐3′ (*Zfhx3* reverse), 5′‐TGCCCGAATGCAAAGGTCTT‐3′ (*Ar* forward), 5′‐TTGGCGTAACCTCCCTTGAAA‐3′ (*Ar* reverse), 5′‐TTTGAAGATTCAGGCGTTATCCG‐3′ (*Fkbp5* forward), 5′‐GGTGGACTTTTACCGTTGCTC‐3′ (*Fkbp5* reverse), 5′‐GCTGTCTTGCTTTGGAGGTTC‐3′ (*Tmprss2* forward) and 5'‐GACAATGTGCTACCCCGTCA‐3′ (*Tmprss2* reverse).

### 2.4 Construction of luciferase reporter plasmids

The promoter‐luciferase reporter plasmid pZFHX3‐Luc was constructed and named pATBF1‐Luc1 in our previous study.^16^ Mutant for ARE6 in the pZFHX3‐Luc was prepared by site‐directed mutagenesis with primers 5′‐TTCTCCCGAGGAAGTGTTCGCCTCATGCT‐3′ and 5′‐GAACACTTCCTCGGGAGAAAAAGGGATGC‐3′, and mutants for ARE1 and ARE1 plus ARE6 in pZFHX3‐Luc were prepared by synthesizing the promoter DNA fragments by Sangon and cloning them into the pGL3‐Basic vector (Promega). The three promoter‐reporter mutant plasmids were named pZFHX3‐Luc‐mARE1, pZFHX3‐Luc‐mARE6 and pZFHX3‐Luc‐mARE(1,6), respectively.

### 2.5 Luciferase reporter assay

C4‐2B cells were transiently transfected with indicated *ZFHX3* plasmids and pRL‐TK Renilla luciferase plasmid (Promega) using the Lipofectamine 2000 reagent (Invitrogen). Forty‐eight hours after transfection, including 24‐h R1881 treatment before cell collection, luciferase activities were determined using the Dual‐Luciferase Reporter Gene Assay Kit (Promega). The luciferase activity was normalized by the Renilla luciferase activity in each sample. Experiments were performed in quadruplication and repeated twice.

### 2.6 Chromatin immunoprecipitation (ChIP) assay

C4‐2B cells were grown in a hormone‐free medium for 1 day or 3 days and treated with 1 nM R1881 for 6 h or 12 h. The SimpleChIP Enzymatic Chromatin IP Kit (Magnetic Beads; Cell Signaling) was used to pull down DNA‐protein complexes according to the manufacturer's instructions. Briefly, cells were cross‐linked with 1% formaldehyde for 10 min, quenched with glycine at room temperature for 5 min, and collected and digested with micrococcal nuclease for 20 min at 37°C. Digestion reactions were stopped by adding 0.5 M EDTA. Nuclear pellets were collected and incubated with protease inhibitors in the ChIP buffer for 10 min on ice. After sonication, chromatin extracts were immunoprecipitated using an anti‐AR antibody (06–680‐AF488, Millipore, Billerica, MA) or IgG.

ChIP products were detected by regular PCR using the following primers: 5′‐ACTCAGGCCAATTCAGCTCCA‐3′ (P1 forward), 5′‐GTGCACCCTTCCGGGTTCTT‐3′ (P1 reverse), 5′‐CTGTCTAAACCCGCTGTACTGT‐3′ (P2 forward), 5′‐CTTACCCACTCTCCAAGCCAG‐3′ (P2 reverse), 5′‐TCTGGCTTGGAGAGTGGGTA‐3′ (P3 forward), 5′‐AAGGCAATTCTTCCCTCGCA‐3′ (P3 reverse), 5′‐GCAGAAGTTGCCAATTCCCT‐3′ (P4 forward), 5′‐GTCAAGCCTGCCTTTGTTCC‐3′ (P4 reverse), 5′‐AGATCACCTGCCTGTGGATT‐3′ (P5 forward), 5′‐TCTGCCCTAATACTGCCACTG‐3′ (P5 reverse), 5′‐GGACAGTGGCAGTATTAGGGC‐3′ (P6 forward), 5′‐ACAGGGGACAACCTCGTAAT‐3′ (P6 reverse), 5′‐CAGCAGGCCTTACCTATCCC‐3′ (P7 forward), 5′‐GGCACAAGCCCAATTCAGTC‐3′ (P7 reverse), 5′‐CCTGTTCTTGGGCCTGAAGT‐3′ (P8 forward), 5′‐CCTGTTGGGTACAGACAGCC‐3′ (P8 reverse), 5′‐GATTCCCTGGAGGCAGTCTT‐3′ (P9 forward), 5′‐CTCGGGAGAAAAAGGGATGCT‐3′ (P9 reverse), 5′‐GGGATGTGATGGTTTTCACC‐3′ (P10 forward), 5′‐CCGATTCTCACAGCACAGAA‐3′ (P10 reverse), 5′‐AGGAGCCTGGAGGCTTACAT‐3′ (P11 forward) and 5′‐GAGCCAATGTGGACAGGAAT‐3′ (P11 reverse).

### 2.7 Extraction of cytoplasmic and nuclear proteins

The Nuclear Protein Extraction Kit (Solarbio) was used to extract cytoplasmic and nuclear proteins. LNCaP and C4‐2B cells grown in complete medium or hormone‐free medium were washed with PBS, harvested with a scraper and collected via centrifugation. Cell pellets were resuspended in 200 μl of PMSF‐supplemented cytoplasmic protein extraction reagent, vortexed for 15 s at maximum speed and incubated in an ice bath for 10 min. Then, the cell lysates were vortexed for 10 s at full speed and centrifuged at 14,000 × *g* at 4°C for 10 min. Cytoplasmic proteins were in the supernatant and collected. The remaining nuclear pellets were added 50 μl of PMSF‐supplemented nuclear protein extraction reagent, vortexed for 15 s at maximum speed, incubated in an ice bath for 10 min and vortexed again for 10 s at full speed. The solution was then centrifuged at 14,000 × *g* at 4°C for 10 min, and nuclear proteins in the supernatant were collected for analysis.

### 2.8 CHX assay

C4‐2B cells were seeded into 12‐well plates at a density of 2 × 10^5^ cells per well. After 24 h, cells were treated with 100 μg/ml of cycloheximide (CHX) with and without 10 μM enzalutamide treatment. At 0, 2, 4 and 6 h after CHX treatment, total proteins were collected, separated by SDS‐PAGE and subjected to Western blotting for ZFHX3 and α‐tubulin. Band intensities were quantified using the ImageJ program (NIH, Bethesda, MD). ZFHX3 protein level was normalized by α‐tubulin level for each time point CHX treatment.

### 2.9 Detection of Zfhx3 expression in mouse prostates after castration

Mouse experiments were approved by the Institutional Animal Care and Use Committee at the Southern University of Science and Technology. Twelve‐week‐old male C57BL/6 mice were purchased from Charles River, and castration was performed as described in our previous study.^23^ One and 14 days after castration, mice were euthanized, and prostates were surgically isolated as previously described.^23^

Immunohistochemistry (IHC) staining was performed to detect protein expression with Zfhx3 antibody (1:2000, PD010, MBL, Nagoya, Japan) and Ar antibody (1:400, 06–680‐AF488, Millipore). Formalin‐fixed, paraffin‐embedded mouse prostates were sectioned at 4 μm, deparaffinized in xylene, rehydrated in graded ethanol (100%–75%) and boiled in 10 mM citrate buffer (pH 6.0) for 5 min (for Zfhx3) or 3 min (for Ar) using a pressure cooker. After treatment with 3% H_2_O_2_ for 10 min, tissue sections for Zfhx3 were blocked with blocking buffer (20 mM HEPES, 1% bovine serum albumin and 135 mM NaCl) and incubated with the anti‐ZFHX3 antibody at room temperature for 1 h. Sections for Ar were blocked with 10% normal goat serum and incubated with the anti‐AR antibody at 4°C overnight. Sections were then incubated with EnVision PolymerHRP secondary antibodies (MXB Biotechnologies) at room temperature for 2 h for Zfhx3 and 30 min for Ar, counterstained with DAB‐chromogen and haematoxylin (MXB Biotechnologies), dehydrated in ethanol and mounted. Slides were scanned using the Aperio VERSA 8 Scanner System (Leica, Wetzlar, Germany). Image‐Pro Plus (NIH) was used to determine the integral optical density (IOD) of the positively stained signal (brown) and area of haematoxylin‐stained signal (blue) for each slide. The IOD/mm^2^ of the haematoxylin‐stained area was then calculated and compared between groups.

### 2.10 Bioinformatic and statistical analyses

The TCGA prostate adenocarcinoma dataset was downloaded from the UCSC Xena public data hub (http://xenabrowser.net/). The dataset contained 499 PCa cancers with the following information: Gleason score, copy number variations (CNVs) estimated by the Genomic Identification of Significant Targets in Cancer (GISTIC) analysis, disease‐free survival status and genome‐wide mRNA expression data. One of the 499 PCa samples did not have mRNA expression data and was excluded for analysis. Some of the PCa samples had information available for their corresponding normal prostate tissues. In the pre‐ranked gene list based on fold changes of RSEM normalized counts, gene set enrichment analysis (GSEA) was performed using the OmicStudio tools (https://www.omicstudio.cn/tool) to ascertain relationships between *ZFHX3* mRNA levels and existing hallmark gene sets. An adjusted *P*‐value of 0.05 was set as the cut‐off point for significant enrichment.

A heatmap was built using the R package, in which the PCa samples were divided into two groups (i.e., *ZFHX3* higher and *ZFHX3* lower) by the median *ZFHX3* expression level. AR activities were defined by the sum of expression levels of 27 genes indicative of AR activities, as established in a previous study.^24^ The expression level of a gene was indicated by the Z‐score of log_2_ (RSEM normalized count +1) relative to that in all PCa samples.

The Pearson correlation analysis was conducted to determine the linear correlation between *ZFHX3* mRNA levels and AR activity scores using GraphPad Prism 6 (GraphPad software).

The differences between rates of *ZFHX3* deletion at one and both copies in PCa samples with different Gleason scores were tested using Fisher's exact test. The disease‐free survival was analysed using the Kaplan‐Meier analysis, and the statistical parameters were calculated using a log‐rank test.

All in vitro experiments were repeated at least twice. Statistical analysis was based on 3 or 4 replicates of one experiment. All experimental readings are expressed as mean ± SD. For statistical comparison between two groups, a two‐tailed Student's *t*‐test was performed. For statistical comparison of more than two groups, one‐way ANOVA or two‐way ANOVA was performed. All statistical analyses were conducted using GraphPad Prism 6. *p* values <0.05 were considered statistically significant.

## 3 RESULTS

### 3.1 Androgen‐AR signalling increases ZFHX3 expression at both protein and mRNA levels

**FIGURE 1:**
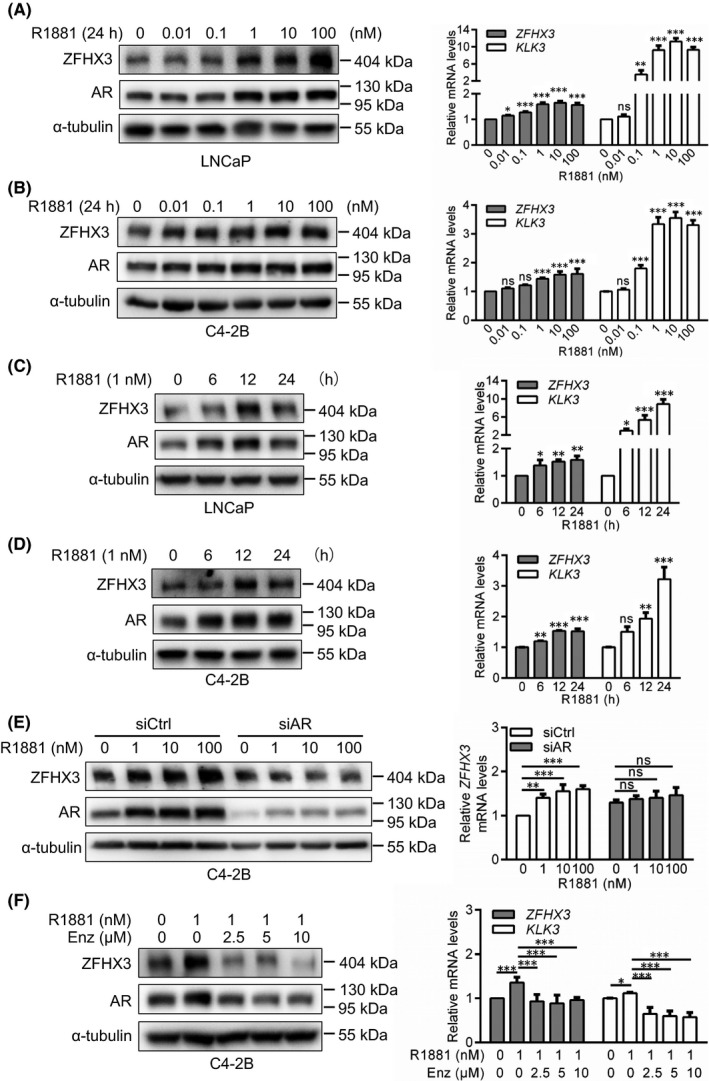
Androgen/AR signalling increases ZFHX3 expression at both mRNA and protein levels in PCa cells. (A–E) Detection of ZFHX3 protein and *ZFHX3* mRNA by western blotting and real‐time qPCR, respectively, along with AR and α‐tubulin as controls in western blotting and *KLK3* as a control in qPCR, in LNCaP (A, C) and C4‐2B (B, D, E) cells cultured in the hormone‐free medium for 24 h and then treated with R1881 at indicated concentrations for 24 h (A, B, E) or 1 nM for indicated times (C, D), with (E) and without the knockdown of *AR* (A–D). Transfection of siRNAs was for 24 h before R1881 treatment. siCtrl, control siRNA; siAR, AR siRNA. (F) In C4‐2B cells cultured in complete medium supplemented with R1881 at 1 nM and enzalutamide at indicated concentrations for 24 h, expression of ZFHX3, AR, α‐tubulin, and *KLK3* was detected as in panels A‐D. ns, not significant; *, *p* < 0.05; **, *p* < 0.01; ***, *p* < 0.001

To test whether androgen modulates ZFHX3 expression, we determined ZFHX3 expression in AR‐positive prostate cancer cell line LNCaP and its derivative C4‐2B under the treatment of the R1881 synthetic androgen.^25^ Cells were incubated in a hormone‐free medium (i.e., phenol red‐free medium supplemented with 5% charcoal‐stripped serum) for 24 h. R1881 treatment caused a dose‐dependent increase in ZFHX3 expression at both protein and mRNA levels in LNCaP and C4‐2B cells (Figure 1A,B). Treatment of the same two cell lines with 1 nM R1881 also increased ZFHX3 expression in a time‐dependent manner (Figure 1C,D). As expected, the R1881 treatment increased the mRNA level of *KLK3*, a classical AR target gene also known as *PSA* (Figure 1A–D).

To further evaluate the role of the androgen/AR signalling in ZFHX3 expression, we knocked down the *AR* by siRNA in C4‐2B cells treated with varying concentrations of R1881 for 24 h in a hormone‐free medium and analysed ZFHX3 expression. *AR* silencing, which was confirmed by western blotting, eliminated the induction of ZFHX3 by R1881 at both protein and mRNA levels (Figure 1E). Interestingly, AR knockdown increased the mRNA level of *ZFHX3* in the absence of R1881 (Figure 1E).

We also applied enzalutamide, an AR antagonist that binds to AR to block its nuclear translocation and transactivation activity,^26^, ^27^ to C4‐2B cells cultured in R1881‐containing complete medium and analysed ZFHX3 expression (Figure 1F). Enzalutamide significantly reduced R1881‐induced ZFHX3 expression at both protein and mRNA levels (Figure 1F). As a control, the mRNA level of *KLK3* was increased by R1881 treatment and decreased by enzalutamide treatments (Figure 1F).

These results suggest that androgen induces ZFHX3 expression via the binding of AR to *ZFHX3* promoter and subsequent transcriptional activation.

### 3.2 Androgen‐induced ZFHX3 expression depends on the binding of AR to the *ZFHX3* promoter

**FIGURE 2:**
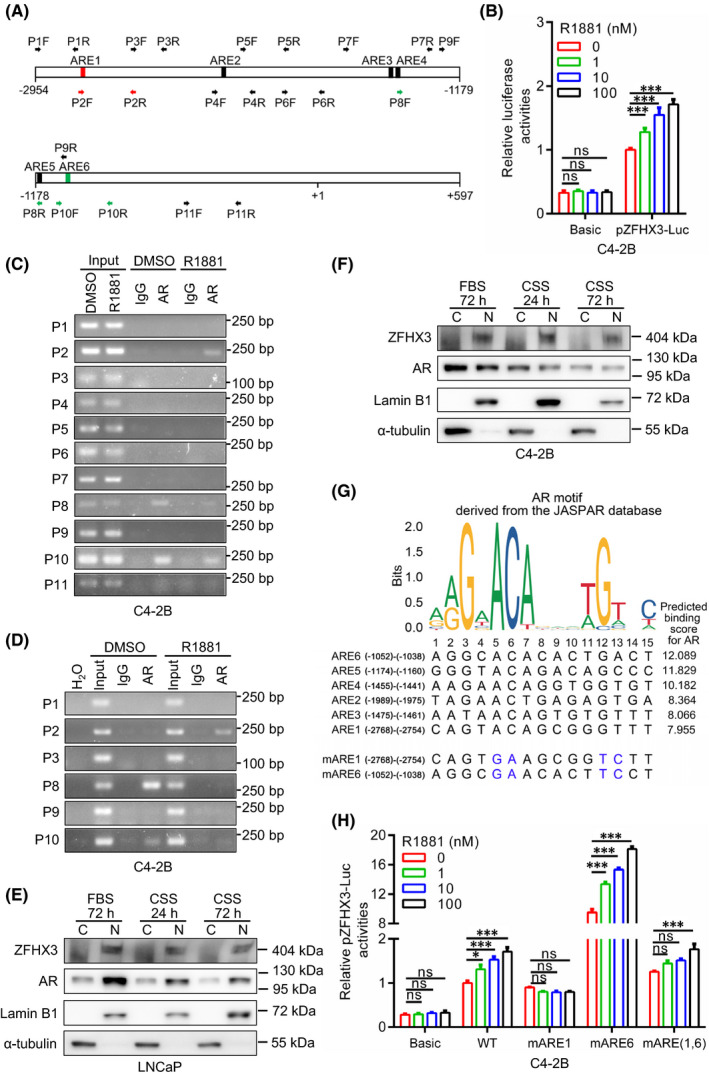
Androgen‐induced *ZFHX3* transcription upregulation involves AR’s binding to *ZFHX3* promoter in PCa cells. (A) Schematic of *ZFHX3* promoter region from −2954 to +597 relative to its transcriptional initiation site. Rectangles indicate the 6 potential androgen‐responsive elements (ARE1‐ARE6), and horizontal arrows indicate PCR primers’ locations for ChIP‐PCR (P1F‐P11R). Red and green arrows correspond to R1881‐enhanced and ‐attenuated, respectively, AR‐bound regions revealed by ChIP‐PCR. (B) Relative luciferase activity of the *ZFHX3* promoter‐reporter plasmid (pZFHX3‐Luc) in C4‐2B cells undergone hormone depletion for 24 h followed by R1881 treatment for 24 h at 1, 10 and 100 nM. (C) Detection of AR‐bound *ZFHX3* promoter DNA in C4‐2B cells using ChIP‐PCR. Cells were pre‐cultured in a hormone‐free medium for 24 h and then treated with R1881 at 1 nM for 6 h before harvesting for ChIP. (D) Confirmation of increased AR binding to ARE1 (P1) and decreased binding to ARE6 (P10) of *ZFHX3* after R1881 treatment in C4‐2B cells with extended hormone depletion (72 h) followed by R1881 treatment at 1 nM for 12 h. (E, F) Detection of AR and ZFHX3 in the nucleus and the cytoplasm by Western blotting in LNCaP (E) and C4‐2B (F) cells cultured in complete medium with 10% FBS or in hormone‐free medium (phenol red‐free medium containing 5% charcoal‐stripped FBS, CSS) for 24 or 72 h. Lamin B1 and α‐tubulin serve as nuclear and cytoplasmic markers, respectively. (G) Comparison of conserved AR binding sequence (top), as defined in the JASPAR database, to the predicted 6 ARE sequences in ZFHX3 (middle). Predicted AR binding scores are shown at the right. Mutants for the first ARE (mARE1) and the sixth ARE (mARE6), with mutated conserved nucleotides in blue, are shown at the bottom. (H) Relative luciferase activities of pZFHX3‐Luc, pZFHX3‐Luc‐mARE1, pZFHX3‐Luc‐mARE6 and pZFHX3‐Luc‐mARE(1,6) in C4‐2B cells with 24 h hormone depletion and subsequent 24 h R1881 treatment at 1, 10 and 100 nM. C, cytoplasmic; N, nuclear; ns, not significant; *, *p* < 0.05; ***, *p* < 0.001

To determine whether androgen‐induced ZFHX3 expression is indeed mediated by the binding of AR to the *ZFHX3* promoter, we analysed the promoter sequence of *ZFHX3* from −2954 to +597 relative to its transcriptional initiation site by the online software JASPAR CORE (http://jaspar.genereg.net/), in which the consensus AR binding sequences are based on ChIP‐Seq studies.^28^ A total of six potential AR binding sites were identified based on the binding scores, which were named ARE1‐ARE6 (Figure 2A).

Then, the pZFHX3‐Luc promoter‐luciferase reporter plasmid for *ZFHX3*, which contains the promoter sequence of *ZFHX3* in Figure 2A, was transfected into C4‐2B cells, and its promoter activity was determined under R1881 treatments in a hormone‐free medium (Figure 2B). The pZFHX3‐Luc reporter plasmid showed a significantly higher promoter activity when compared to the pGL3‐Basic control. More importantly, androgen increased the promoter activity in a dose‐dependent manner (Figure 2B), which is consistent with the induction of ZFHX3 expression by R1881.

To further examine the role of AR in *ZFHX3* transcription, including whether AR has different binding patterns for different AREs under R1881 treatment, we performed a ChIP‐PCR assay in C4‐2B cells undergone 24‐h hormone starvation and subsequent 6‐h R1881 treatment (Figure 2C). PCR primers were designed to cover all six AREs, the sequences between AREs, and the region next to the transcription initiation site in the cloned *ZFHX3* promoter (Figure 2A). The P2 primer pair, which span ARE1, showed a clear R1881‐induced binding (Figure 2C), which is consistent with R1881‐induced patterns of *ZFHX3* transcription and pZFHX3‐Luc promoter activity. Primers pairs P8 and P10, which span AREs 5 and 6, showed an unexpected pattern of AR binding, as the binding occurred before R1881 treatment and was weakened by R1881 (Figure 2C). No binding of AR was detected for other primer pairs (Figure 2C).

We extended hormone depletion time from 24 h to 72 h and R1881 treatment time from 6 h to 12 h and performed ChIP‐PCR for selected primer pairs again. The pattern of AR binding was unchanged, as the AR‐bound P2 fragment was increased by R1881, P8 and P10 fragments were decreased, and other fragments were not affected (Figure 2D). This finding confirms that different AREs in the *ZFHX3* promoter have different responses to androgen‐induced AR binding and suggests that ARE1 is likely responsible for R1881‐induced *ZFHX3* upregulation, but ARE6 likely has an opposite effect.

The binding of AR to P8 and P10 fragments of the *ZFHX3* promoter without androgen indicates that some AR molecules exist in the nucleus even without androgen. To test whether this is true, we grew LNCaP and C4‐2B cells in hormone‐free medium or complete medium for 24 and 72 h, extracted cytoplasmic and nuclear proteins and analysed the expression of AR and ZFHX3 by Western blotting. In the absence of androgen, AR protein was indeed detected in both the cytoplasm and the nucleus. In contrast, ZFHX3 protein was primarily detected in the nucleus (Figure 2E,F), although AR protein was increased in the cytoplasm and decreased in the nucleus (Figure 2E,F). Therefore, even without androgen, some AR proteins still exist in the nucleus in PCa cells.

### 3.3 AR‐bound ARE1 and ARE6 have opposing effects on *ZFHX3* transcription

We further characterized ARE1 and ARE6 for their opposite effects on R1881‐induced *ZFHX3* transcription. In the pZFHX3‐Luc reporter plasmid, ARE1, ARE6, or both ARE1 and ARE6 were mutated at the consensus ARE nucleotides, and the mutant reporter plasmids were then analysed for promoter activities (Figure 2G).

Mutation of ARE1 not only decreased *ZFHX3* promoter activity but also prevented the induction of promoter activity by R1881 (Figure 2H). It appeared that R1881 further decreased the promoter activity of pZFHX3‐Luc‐mARE1. On the other hand, mutation of ARE6 dramatically increased *ZFHX3* promoter activity while maintaining the promoter's response to R1881 (Figure 2H). When both ARE1 and ARE6 were mutated, the promoter's activities were somewhat similar to that of the wildtype, maintaining a modest promoter activity and androgen responses (Figure 2H). The results further indicate that AR‐bound ARE1 and ARE6 have opposite effects on *ZFHX3* transcription, with ARE1 increasing but ARE6 decreasing *ZFHX3* transcription in AR‐positive PCa cells.

### 3.4 Inhibition of AR activity by enzalutamide causes ZFHX3 protein degradation without apparent change in *ZFHX3* mRNA level

**FIGURE 3:**
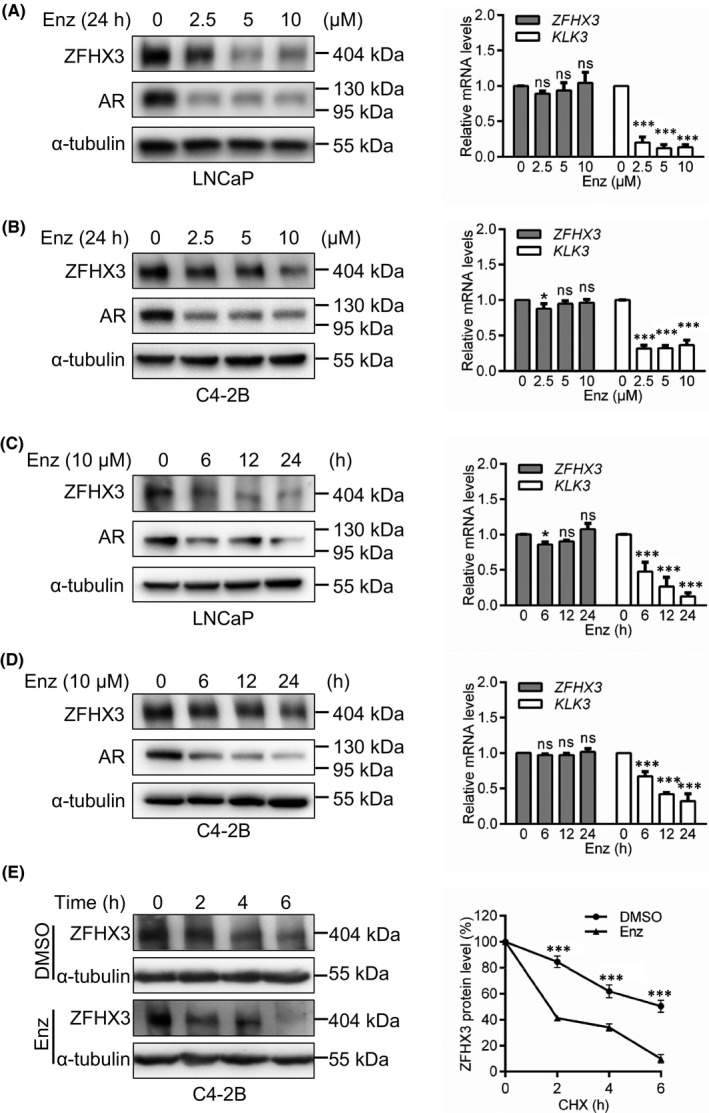
AR antagonist enzalutamide causes the degradation of ZFHX3 protein without affecting *ZFHX3* transcription in PCa cells. (A–D) Detection of ZFHX3 protein by Western blotting and *ZFHX3* mRNA by real‐time qPCR, with AR and α‐tubulin as controls in Western blotting and *KLK3* as a control in qPCR, in LNCaP (A, C) and C4‐2B (B, D) cells cultured in complete medium supplemented with enzalutamide at indicated concentrations for 24 h (A, B) or 10 μM for indicated times (C–D). (E) Determination of ZFHX3 half‐life by the CHX assay. In C4‐2B cells cultured in the complete medium containing cycloheximide (100 μg/ml) and enzalutamide (10 μM), Western blotting was used to detect proteins (left) and the ImageJ program used to quantify band intensities (right). Band intensity ratios below each lane of the western blot in panel E were the average from three independent experiments. ns, not significant; *, *p* < 0.05; ***, *p* < 0.001

To further test whether enzalutamide decreases ZFHX3 protein level without significantly affecting *ZFHX3* mRNA expression, as suggested by the result in Figure 1F, we treated LNCaP and C4‐2B cells cultured in complete medium with enzalutamide at varying concentrations for 24 h or 10 µM for varying times. Enzalutamide indeed decreased ZFHX3 protein level in a dose‐ and time‐dependent manner but did not cause a noticeable decrease in *ZFHX3* mRNA level. The protein level of AR and the mRNA level of *KLK3*, an AR target gene, were decreased by enzalutamide treatment as expected (Figure 3A–D), suggesting an untypical regulation of ZFHX3 by AR.

To test whether enzalutamide decreases ZFHX3 protein level via protein degradation, we measured the half‐life of ZFHX3 protein in C4‐2B cells treated with cycloheximide (CHX) for different times. The CHX assay demonstrated that enzalutamide significantly decreased the half‐life of ZFHX3 protein (Figure 3E), suggesting that enzalutamide causes ZFHX3 protein degradation.

### 3.5 Androgen ablation in mice decreases Zfhx3 expression in mouse prostates

**FIGURE 4:**
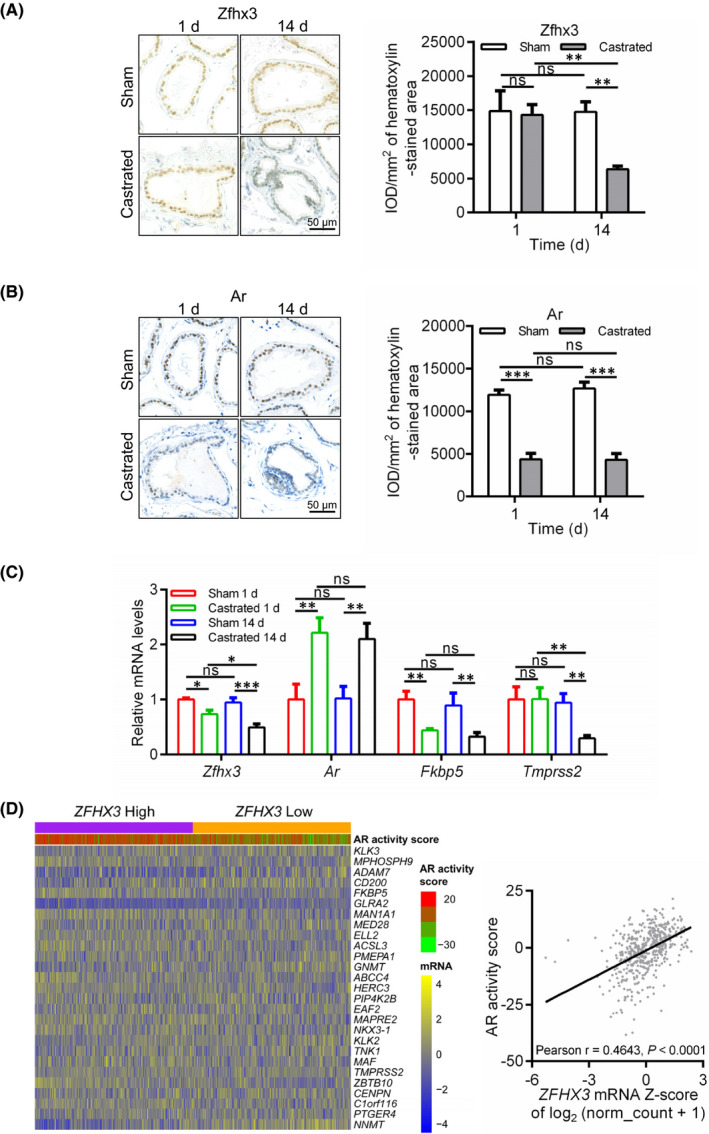
Upregulation of ZFHX3 by the AR signalling also occurs in normal prostates and prostate cancers. (A–B) Detection of Zfhx3 (A) and Ar (B) proteins by IHC staining in mouse prostates after castration for 1 day and 14 days. Protein signal intensities were quantified as integrated optical density (IOD), and IOD/mm^2^ of the haematoxylin‐stained area was used to indicate a protein's expression level. (C) Detection of *Zfhx3*, *Ar* and Ar target genes *Fkbp5* and *Tmprss2* by real‐time qPCR in the same mouse prostates as in A and B. *N* = 3 mice for each group. (D) Expression of *ZFHX3* is positively correlated with AR activity in human PCa specimens, as revealed by the heatmap of the 27 genes indicative of AR activity in tumours with higher and lower *ZFHX3* expression (panel at left) and Pearson correlation analysis (panel at right). RNA‐sequencing data used in these analyses were from the PRAD dataset in the TCGA database. ns, not significant; *, *p* < 0.05; **, *p* < 0.01; ***, *p* < 0.001

To determine whether the regulation of ZFHX3 expression by the androgen‐AR signalling also occurs in vivo, we castrated 12‐week‐old C57BL/6 mice to deplete androgen and detected Zfhx3 expression in mouse prostates. IHC staining demonstrated that the Zfhx3 protein was primarily expressed in the nucleus of luminal cells with a uniform staining intensity; Zfhx3 expression decreased at 14 days after castration but did not show a noticeable change at 1 day after castration, as indicated by the integrated optical density (IOD) (Figure 4A). As expected, the Ar protein was exclusively expressed in the nucleus of luminal cells with a nonuniform staining intensity; and Ar expression decreased at both 1 day and 14 days after castration (Figure 4A,B).

Real‐time qPCR demonstrated that at both d1 and d14 after castration, *Zfhx3* mRNA level decreased, and Ar mRNA level was increased, which is considered negative autoregulation.^29^, ^30^ As expected, the mRNA levels of known AR target genes *Fkbp5* and *Tmprss2* were decreased by castration (Figure 4C). These results further indicate that the androgen‐AR signalling induces the expression of ZFHX3 in prostate cells.

### 3.6 ***ZFHX3*** **mRNA levels positively correlate with AR activities in human PCa specimens**

To evaluate whether transcriptional regulation of *ZFHX3* by the androgen‐AR signalling also occurs in human prostate cells, we collected RNA‐sequencing data of PCa samples from the TCGA database. We then analysed *ZFHX3* expression levels and AR activities defined by the sum of expression levels of 27 genes that showed robust activation or inhibition of expression upon androgen stimulation.^24^ The 498 PCa samples with mRNA data were stratified into two groups according to the median level of *ZFHX3* expression: *ZFHX3*‐high and *ZFHX3*‐low. The heatmap demonstrated that tumours with a higher *ZFHX3* level also had a higher AR activity (Figure 4D, *left*). Pearson correlation analysis further confirmed the positive correlation between *ZFHX3* expression levels and AR activity scores regardless of tumour stage, grade, metastasis or age (Figure 4D, *right*; data not shown). Therefore, induction of *ZFHX3* transcription by the androgen‐AR signalling also occurs in human prostatic epithelial cells.

### 3.7 Alterations of *ZFHX3* in human PCa and their potential impacts

**FIGURE 5:**
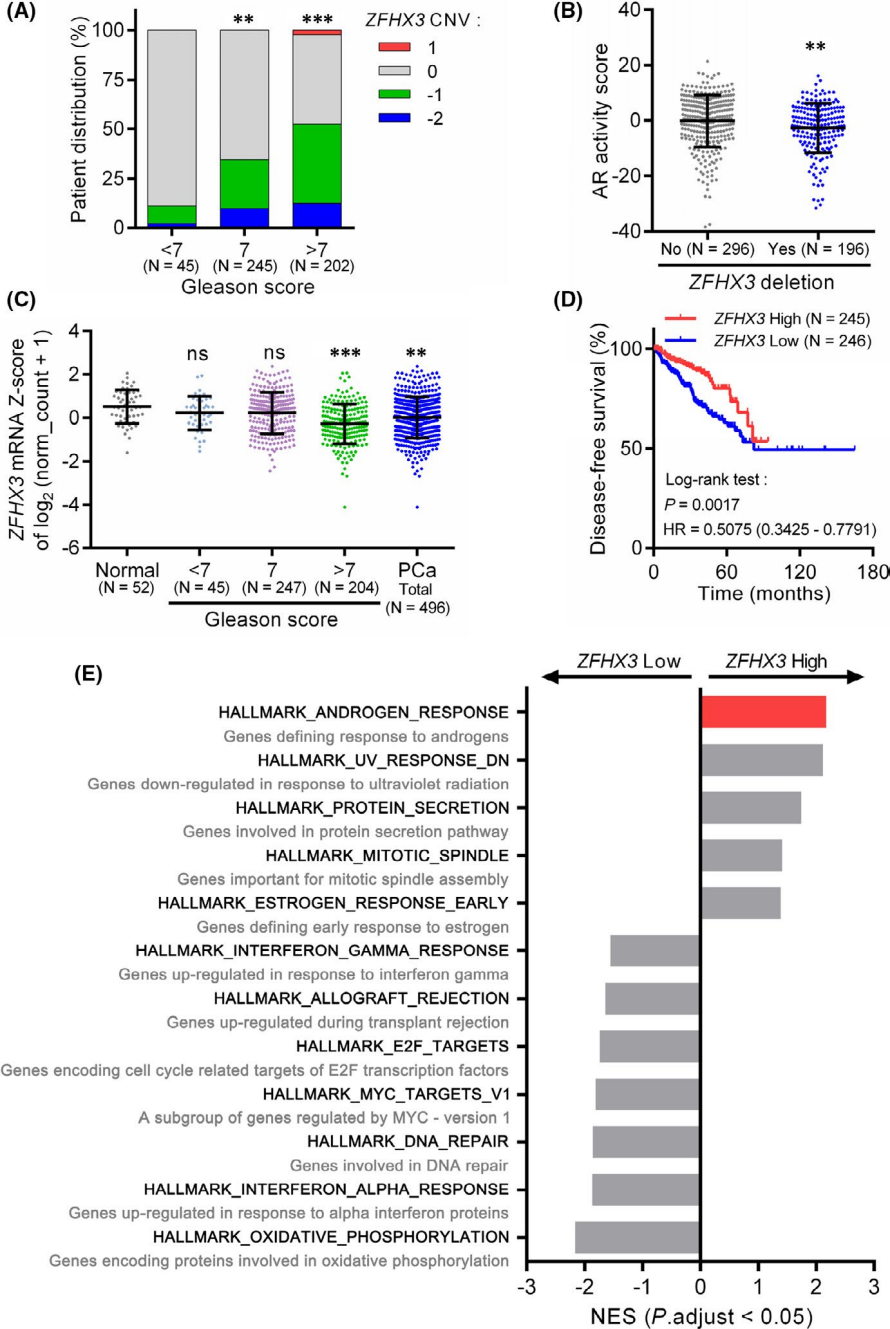
Alterations of *ZFHX3* in human PCa and their potential impacts. (A) Distribution of *ZFHX3* copy number variations (CNVs) in human PCa specimens with different Gleason scores. (B) Deletion of *ZFHX3* at one or two copies correlates with AR activity scores defined by a panel of 27 genes. (C) *ZFHX3* mRNA expression level, as defined by the Z‐score of log_2_(RSEM normalized count +1) relative to all PCa samples, is reduced in PCa; and the reduction is more frequent in PCa with higher Gleason scores. (D) Downregulation of *ZFHX3* in PCa correlates with worse disease‐free survival in PCa patients, as determined by the Kaplan‐Meier analysis. (E) Enrichment of hallmark gene sets in *ZFHX3*‐high and *ZFHX3*‐low PCa specimens, with the *x*‐axis representing normalized enrichment scores (NES). HR, hazard ratio; ns, not significant; **, *p* < 0.01; ***, *p* < 0.001

Using the TCGA PCa dataset, we evaluated the status of *ZFHX3* and its potential clinical relevance in PCa. We found that deletion of one or both copies of *ZFHX3* was more frequent in PCa samples with higher Gleason scores (Figure 5A). Interestingly, deletion of *ZFHX3* significantly correlated with reduced AR activities defined by the sum of 27 genes’ expression levels that show robust expression activation or inhibition upon androgen stimulation^24^ (Figure 5B), supporting a role of ZFHX3 in AR function.

The *ZFHX3* mRNA levels, as indicated by the Z‐scores of log_2_(RSEM normalized count +1) relative to that of all PCa samples, were lower in PCa samples, particularly those with Gleason scores greater than 7 (Figure 5C). We also tested the potential relationship between *ZFHX3* expression and disease‐free survival in human PCa. Kaplan‐Meier curve with log‐rank analysis demonstrated that patients with lower *ZFHX3* expression levels had poorer disease‐free survival (Figure 5D), implicating that decreasing *ZFHX3* expression could aggravate the progression of PCa.

To explore the biological functions of ZFHX3, we divided the 498 PCa samples with mRNA data into two groups, *ZFHX3*‐high and *ZFHX3*‐low, according to the median *ZFHX3* mRNA level, as indicated by the Z‐score of log_2_(RSEM normalized count +1). The gene set enrichment analysis (GSEA) was then performed using the pre‐ranked list of genes based on fold changes. The top five enriched hallmarks included androgen response genes, genes downregulated in response to UV radiation, protein secretion, mitotic spindle and early response to oestrogen. Those enriched in the *ZFHX3*‐low group included interferon‐gamma response, allograft rejection, E2F targets, MYC‐regulated genes, DNA repair, interferon‐alpha response, and oxidative phosphorylation (Figure 5E). The hallmark for androgen response included 100 genes defining response to androgens. Among the *ZFHX3*‐high group, the androgen‐responsive genes had the highest normalized enrichment score (NES), further supporting a role of ZFHX3 in AR function (Figure 5E).

## 4 DISCUSSION

Our findings in this study establish a regulatory relationship between the androgen/AR signalling and the *ZFHX3* transcription factor; that is, androgen/AR signalling upregulates *ZFHX3* in PCa cells. This conclusion is supported by multiple lines of evidence, including time‐ and dose‐dependent induction of *ZFHX3* mRNA and ZFHX3 protein by the synthetic androgen R1881 (Figure 1), activation of the *ZFHX3* promoter by R1881 and dynamic binding of AR to multiple androgen‐responsive elements (AREs) in the *ZFHX3* promoter (Figure 2) in LNCaP and C4‐2B cells. Notably, the transcriptional regulation of *ZFHX3* by the androgen/AR signalling also occurs in vivo, as castration‐mediated androgen depletion in mice downregulated *ZFHX3* mRNA and protein levels in the prostate and the *ZFHX3* mRNA levels positively correlated with AR activities in human PCa specimens (Figure 4). It is noteworthy that this study provides for the first time that in mouse prostates, Zfhx3 protein is primarily localized in the nucleus of luminal cells with a uniform staining intensity, as demonstrated by IHC staining (Figure 4A).

Androgen is the primary steroid hormone that drives normal epithelial homeostasis and carcinogenesis in the prostate, which has also been demonstrated in LNCaP and C4‐2B cells, as R1881 promotes cell proliferation in both cell lines.^31^, ^32^ Androgen deprivation therapy is thus widely used to treat patients with prostate cancer. Meanwhile, ZFHX3 has also been shown to modulate both normal development and carcinogenesis of the prostate, as *ZFHX3* is frequently mutated in advanced human prostate cancer and loss of Zfhx3 in mouse prostates causes or promotes prostatic carcinogenesis.^11^, ^13^, ^14^, ^15^ Our bioinformatic analyses in this study provide evidence for the copy number loss and downregulation of *ZFHX3* in PCa, particularly those with higher Gleason scores (Figure 5A,C). In addition, downregulation of *ZFHX3* was correlated with worse disease‐free survival in patients with PCa (Figure 5D), further supporting the role of ZFHX3 in human PCa. Transcriptional activation of *ZFHX3* by the androgen/AR signalling thus suggests that the proper function of AR depends on the existence of ZFHX3 in prostate epithelial cells. The role of ZFHX3 in AR function is further supported by the correlation between *ZFHX3* deletion and reduced AR activities (Figure 5B) and the enrichment of androgen‐responsive genes in PCa samples expressing higher levels of *ZFHX3* (Figure 5E). Whether and how AR and ZFHX3 functionally cooperate is a meaningful question that has not been addressed. ZFHX3 could be involved in the balance between cell proliferation and differentiation induced by androgen in normal AR‐positive epithelial cells.

Autoregulation of *AR* mRNA and protein is an evolutionary conserved regulatory mechanism.^33^ While the autoregulation of AR appears to be negative in most AR‐expressing rat tissues and human cell lines,^29^, ^30^, ^34^ some human cells appear to have a positive AR autoregulation, including PC‐3 and DU 145 PCa cells ectopically expressing AR and osteoblastic cells of Saos‐2.^35^, ^36^, ^37^, ^38^ The multiple exonic AREs could mediate such a contrasting pattern of AR autoregulation in the *AR* gene.^30^, ^36^ In the current study, we found that activation of the androgen/AR signalling by R1881 increased, while inhibition of the signalling by enzalutamide or castration decreased, AR protein levels in LNCaP and C4‐2B cells and mouse prostates (Figures 1,3A–D,4B). In contrast, castration increased *AR* mRNA levels in mouse prostates (Figure 4C). The opposite pattern of autoregulation of AR may correspond to its different functions, that is, enhancing cellular responsiveness to androgen and maintaining homeostasis.

There are multiple potential AREs in the *ZFHX3* promoter. Two of them, ARE1 and ARE6 (Figure 2A), are critical for AR‐mediated *ZFHX3* transcription, as demonstrated by promoter‐reporter assay and ChIP‐PCR (Figure 2). ARE1 acts as a classic ARE for an AR target gene, as androgen induced the binding of AR to ARE1 of *ZFHX3* promoter, the binding activated *ZFHX3* transcriptional activity, and mutation of the ARE1 eliminated androgen‐induced *ZFHX3* promoter activity (Figure 2).

However, ARE6 acts in an opposing manner compared to ARE1. AR bound to ARE6 without androgen, and androgen treatment interrupted the binding (Figure 2C,D). In addition, the androgen‐independent binding of AR to ARE6 represses the *ZFHX3* promoter activity, as mutation of ARE6 dramatically increased *ZFHX3* promoter activity both with and without androgen treatment (Figure 2H). Consistently, *AR* knockdown in the absence of androgen upregulated *ZFHX3* transcription in the same cell lines (Figure 1E). Therefore, it appears that at least in some AR‐positive prostate cancer cells, AR is present in the nucleus even in the absence of androgen, and such androgen‐independent AR binds to the *ZFHX3* promoter to repress its transcription.

Such a unique pattern of androgen‐induced reversal in gene transcription is unusual. Still, it has been reported that the prostate‐specific membrane antigen (*PSMA*) gene (also known *FOLH1*) is transcriptionally upregulated without androgen but repressed when androgen is present due to an enhancer element.^39^, ^40^

Western blotting of cytoplasmic and nuclear proteins showed that some AR molecules were localized in the nucleus without androgen binding (Figure 2E,F). Such a ligand‐independent nuclear localization for AR is somewhat unexpected but demonstrated previously in LNCaP and LNCaP‐derived C4‐2 cell lines by Western blotting and immunofluorescence staining.^41^, ^42^, ^43^ In general, the nuclear receptor AR depends on androgen as its ligand to enter the nucleus and bind to gene promoters, so whether and how some AR molecules can enter into and function in the nucleus without androgen binding is interesting and worth further investigation.

We noticed that when both ARE1 and ARE6 were mutated, androgen treatment still induced a detectable *ZFHX3* promoter activity (Figure 2H), suggesting that one or more AREs within the cloned *ZFHX3* promoter (Figure 2A) also play a role in androgen‐induced *ZFHX3* transcription.

Enzalutamide, however, while preventing the activation of *ZFHX3* transcription by androgen (Figure 3A–D), did not decrease *ZFHX3* transcription as in the transcription of *KLK3*, a classic target gene of AR (Figure 3A–D). Lack of enzalutamide‐induced *ZFHX3* downregulation could be attributed, at least in part, to AR’s binding to ARE6 without androgen. For example, the binding of enzalutamide to AR, while preventing cytoplasmic AR from entering the nucleus and reducing AR protein level, could release androgen‐free nuclear AR from the ARE6 promoter DNA, eliminating the repression of AR activity on ARE6. This speculation remains further tested.

While not decreasing *ZFHX3* mRNA level further as expected, enzalutamide treatment significantly decreased ZFHX3 protein level via excess protein degradation (Figure 3E). Enzalutamide downregulated AR protein as expected (Figure 3). Simultaneous downregulation of both AR and ZFHX3 proteins further suggests the necessity of ZFHX3 for AR function. Such a necessity is also supported by the observation that AR activity scores were lower in PCa samples with *ZFHX3* deletion and higher *ZFHX3* expression correlated with genes indicative of AR activities (Figures 4D,5B).

*ZFHX3* undergoes frequent loss‐of‐function mutation in advanced prostate cancer.^11^ Its deletion is more frequent in PCa specimens with higher Gleason scores (Figure 5A), and loss of Zfhx3 induces or promotes neoplastic lesions in the prostate.^13^, ^14^ In addition, downregulation of *ZFHX3* correlated with lower AR activities and worse disease‐free survival in PCa patients (Figures 4D,5D). On the other hand, ectopic expression of *ZFHX3* decreases colony formation in the 22Rv1 CRPC cell line.^11^ It is thus likely that loss or downregulation of *ZFHX3* enhances the development of castration resistance in PCa. In this regard, whether the downregulation of ZFHX3 protein by enzalutamide affects therapy resistance becomes a valid question. For example, while enzalutamide is effective in treating advanced and metastatic prostate cancer,^27^, ^44^ therapeutic resistance and subsequent recurrence eventually occur after enzalutamide treatment,^45^, ^46^ and over‐activation of the PI3K/Akt pathway is often involved.^47^, ^48^ On the other hand, loss of Zfhx3 in mouse prostates activates the Akt signalling pathway.^13^, ^14^ It thus should be meaningful to determine whether the downregulation of ZFHX3 protein by enzalutamide contributes to its therapeutic resistance.

If downregulation of ZFHX3 indeed contributes to the development of castration resistance, the AR‐ZFHX3 interaction and its downstream target genes could provide an opportunity for developing agents that could overcome resistance to ADT in PCa.

In summary, we found that in prostatic epithelial cells, androgen upregulates the transcription of *ZFHX3* via the binding of AR to specific AREs in the *ZFHX3* promoter, and the regulatory relationship between androgen/AR and *ZFHX3* is valid in both mouse prostates and human prostate cancer specimens. When androgen is absent, some AR molecules in the nucleus bind to an ARE in the *ZFHX3* promoter, and the binding represses *ZFHX3* transcription. Furthermore, the enzalutamide antiandrogen prevents AR from activating *ZFHX3* transcription while causing excess ZFHX3 protein degradation. A dynamic regulatory relationship between the androgen/AR signalling and ZFHX3, both of which modulate prostate cancer development and progression, could have therapeutic implications.

## CONFLICT OF INTEREST

The authors declare that they have no competing interests.

## AUTHOR CONTRIBUTIONS

**Xing Fu:** Conceptualization (equal); Investigation (lead); Writing – original draft (lead). **Zhiqian Zhang:** Writing – review & editing (equal). **Mingcheng Liu:** Investigation (supporting); Methodology (equal). **Juan Li:** Investigation (supporting); Methodology (equal). **Jun A:** Data curation (equal); Software (equal). **Liya Fu:** Project administration (supporting). **Chenyang Huang:** Project administration (supporting). **Jin‐Tang Dong:** Conceptualization (lead); Funding acquisition (lead); Supervision (lead); Writing – review & editing (lead).

## Data Availability

The data that support the findings of this study are available from the corresponding author upon reasonable request.
